# Food-Derived Natural Compounds for Pain Relief in Neuropathic Pain

**DOI:** 10.1155/2016/7917528

**Published:** 2016-11-07

**Authors:** Eun Yeong Lim, Yun Tai Kim

**Affiliations:** ^1^Research Group of Innovative Special Food, Korea Food Research Institute, No. 62, Anyangpangyo-ro, Bundang-gu, Seongnam 13539, Republic of Korea; ^2^Department of Food Biotechnology, Korea University of Science and Technology, 217 Gajeong-ro, Yuseong-gu, Daejeon 34113, Republic of Korea

## Abstract

Neuropathic pain, defined as pain caused by a lesion or disease of the somatosensory nervous system, is characterized by dysesthesia, hyperalgesia, and allodynia. The number of patients with this type of pain has increased rapidly in recent years. Yet, available neuropathic pain medicines have undesired side effects, such as tolerance and physical dependence, and do not fully alleviate the pain. The mechanisms of neuropathic pain are still not fully understood. Injury causes inflammation and immune responses and changed expression and activity of receptors and ion channels in peripheral nerve terminals. Additionally, neuroinflammation is a known factor in the development and maintenance of neuropathic pain. During neuropathic pain development, the C-C motif chemokine receptor 2 (CCR2) acts as an important signaling mediator. Traditional plant treatments have been used throughout the world for treating diseases. We and others have identified food-derived compounds that alleviate neuropathic pain. Here, we review the natural compounds for neuropathic pain relief, their mechanisms of action, and the potential benefits of natural compounds with antagonistic effects on GPCRs, especially those containing CCR2, for neuropathic pain treatment.

## 1. Introduction

The International Association for the Study of Pain (IASP) defines neuropathic pain as pain caused by a lesion or disease of the somatosensory nervous system, which causes unpleasant and abnormal sensation (dysesthesia), an increased response to painful stimuli (hyperalgesia), and pain in response to a stimulus that does not normally provoke pain (allodynia) [1, 2]. This definition of neuropathic pain distinguishes it from other types of pain, including musculoskeletal pain, by restricting its extent to the somatosensory nervous system.

According to previous studies, neuropathic pain affects about 1 in every 10 adults and the economic burden for treating this pain is increasing [3, 4]. Langley and colleagues have described the importance of pain in terms of social impact and have shown that people experiencing neuropathic pain have an economic burden twice that of patients with chronic nonneuropathic pain, in five countries in Western Europe [5, 6].

There are four main types of pharmacological therapies for neuropathic pain: antidepressants, anticonvulsants, opioids, and topical agents. The first-line treatments for neuropathic pain, based on efficacy and safety, include antidepressants (e.g., tricyclic antidepressants [TCAs], serotonin-norepinephrine reuptake inhibitors [SNRIs]) and certain anticonvulsants (e.g., gabapentin, pregabalin, and topical lidocaine) [7]. Opioid analgesics have been recommended as second-line treatments, given their safety; however, they are sometimes used as first choice. Third-line treatments include certain antidepressant medications (e.g., bupropion, citalopram, and paroxetine) and certain anticonvulsants medications (e.g., carbamazepine, lamotrigine, oxcarbazepine, and N-methyl-D-aspartate [NMDA] receptor antagonists). However, these drugs are not completely effective in attenuating neuropathic pain, because of the complexity of this type of pain, and also have side effects, such as sedation, dizziness, edema, and ataxia [8, 9]. For these reasons, there is interest in new agents for relieving neuropathic pain. Although the existing neuropathic pain animal model does not fully represent the human condition, it facilitates studies on nerve injury-induced pain and indicates neuropathic pain mediators. Natural products have been widely used for centuries to treat various diseases and can effectively treat diseases, without causing side effects [10], and may present therapeutic candidates for the development of new drugs to alleviate neuropathic pain.

The causes of neural damage can be diverse; these include diabetic neuropathy, human immunodeficiency virus (HIV) neuropathy, postherpetic neuralgia, drug-induced neuropathy, and traumatic nerve injury. Various neuropathic pain models have been developed, with consideration of different neuroimmune signaling pathways [11]. Tissue and nerve injury-induced hyperexcitability are due to immune cells and the inflammatory mediators that they release. In particular, it is well-known that neuroinflammation, which is a local inflammatory reaction in the nervous system, can lead to the development of neuropathic pain. In addition, ion channels open and close in response to chemical or mechanical signals, and G protein-coupled receptors (GPCRs) also stimulate hyperexcitability. GPCRs regulate ligand-gated and voltage-dependent ion channels and are activated in response to inflammatory mediators that are released by peripheral tissues and immune cells.

In this review, we summarize the processes involved in neuropathic pain development and natural compounds that are useful for neuropathic pain alleviation and further discuss the potential benefits of C-C motif chemokine receptor 2 (CCR2) antagonists for treatment of neuropathic pain.

## 2. Mechanism of Neuropathic Pain

**Figure 1:**
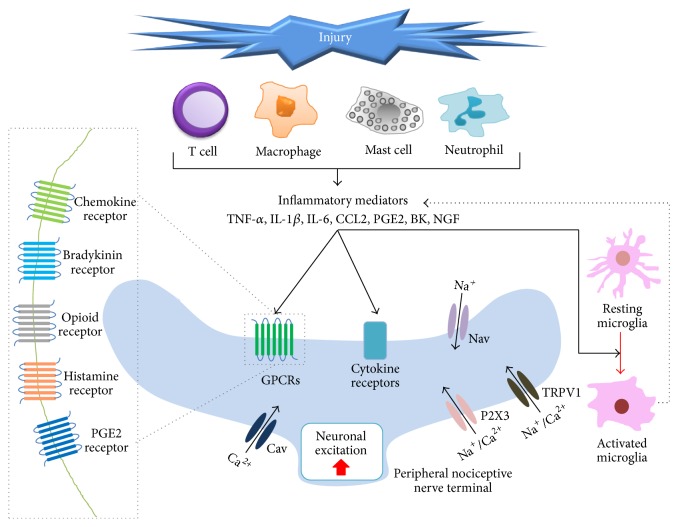
Mechanisms involved in peripheral sensitization in neuropathic pain. Tissue and nerve injury lead to inflammation, including neuroinflammation, via activation of immune cells (T cells, macrophage, mast cell, and neutrophil), by releasing diverse inflammatory mediators, such as TNF-*α*, IL-1*β*, IL-6, CCL2, PGE2, BK, and NGF, in nearby peripheral nociceptive nerve terminal. These mediators act on their respective receptors, including TRPV1, P2X3, cytokine receptors, and G-coupled protein receptors. The activation of these receptors results in upregulation of Ca^2+^ and cyclic AMP, which related to several kinase signaling pathways. Altogether, these mechanisms can lead to neuronal excitation and microglial activation, as well as gene expression changes in sensory neurons. Overactivated microglia can also trigger neuropathic pain via inflammatory mediator-related signaling.

Generally, pain perception involves the following processes: transduction, transmission, modulation, and perception. In brief, nociceptors switch noxious stimulation to nociceptive signals, which are transported into the central nervous system (CNS) along nerve fibers from the site of injury. These nociceptive signals are modulated at synaptic sites and in the CNS by ascending and descending pathways, and we recognize pain [12]. In neuropathic pain, nerve injury alters expression of genes encoding cytokine and chemokine receptors; ion channel expression at the membranes and substances released by immune cells induce nociceptive signaling in the peripheral and central nervous system and ultimately cause the development of neuropathic pain (Figure 1) [13, 14]. Moreover neuroinflammation, that is, immune reaction in the peripheral and CNS, including activation of mast cells and glial cells, may occur due to nerve injury. These changes induce peripheral sensitization, which involves enhanced excitability of the primary afferent nociceptors that convert peripheral stimuli into action potentials that are propagated in the CNS [15]. Thus, peripheral sensory nerve hyperexcitability and central nerve hyperexcitability, involving diverse pathways, are the major reasons for the initiation, development, and maintenance of neuropathic pain [16]. After peripheral nerve injury, microglia in a resting state in the spinal dorsal horn and dorsal root ganglia (DRG) are activated through a series of cellular and molecular changes that are mainly produced by monocyte chemoattractant protein 1/C-C motif chemokine ligand 2 (MCP1/CCL2). This activation is commonly observed among various models of neuropathic pain [17].

### 2.1. Neuroimmune Interactions Are Mediated by Inflammatory Cytokines and Chemokines

Growing evidence indicates that inflammatory and immune responses contribute to neuropathic pain [18]. After nerve injury, immunocompromised mice show alleviation of neuropathic pain as compared with normal mice, in the early stage [19]. In addition, neuroinflammation induced by nervous system injury accompanies a neuroimmune interaction that activates immune cells and leads to development of chronic pain, including neuropathic pain. For instance excessive neuroinflammation, as found in Guillain–Barré syndrome and multiple sclerosis, is associated with neuropathic pain. In this regard, targeting neuroinflammation is also a promising analgesic approach for neuropathic pain [20, 21].

One characteristic of neuropathic pain is infiltration of blood leukocytes by chemokines [22]. Accumulating evidence has indicated that glial cell activation and the resulting expression of proinflammatory immune mediators, such as tumor necrosis factor-*α* (TNF-*α*), interleukin-1*β* (IL-1*β*), and brain-derived neurotrophic factor (BDNF), play critical roles in neuropathic pain [23, 24]. In early life, nerve injury triggers anti-inflammatory responses, such as expression of IL-4 and IL-10 rather than proinflammatory mediators, supporting the supposition that infants rarely experience neuropathic pain [25].

We will describe immune responses focused on immune cells (mast cells, microglia, neutrophils, macrophages, Schwann cells, and T cells) and some of their inflammatory mediators. In a normal state, mast cells have granules that contain a variety of bioactive chemicals. Nervous injury triggers neuroinflammation, which activates mast cells, and activated mast cells in turn release inflammatory factors, such as bradykinin, prostaglandins, histamine, and substance P [26]. Partial sciatic nerve ligation (PSNL) induces mast cell activation and degranulation and neutrophil and macrophage infiltration, which is reversed by treatment with a mast cell stabilizer. This suggests that mast cells are powerful neuropathic pain mediators [27]. Microglia, which are glial cells that are located throughout the CNS, become activated in response to nerve injury and immunological stimuli, including proinflammatory signals released from immune cells, such as mast cells. This interaction between mast cells and microglia regulates peripheral pain signaling. Microglia activation causes pain states by releasing proinflammatory cytokines, chemokines, and proteases [28]. Astrocytes, the most abundant glial cell type in the CNS, also play a major role in pain facilitation and are fundamental contributors to the neuropathic pain involved in neuroinflammation. Therefore, a promising therapeutic target for managing neuropathic pain would be factors that mediate mast and glia cell reactivity.

In a normal state, neutrophils, which are the first leukocytes to arrive at a site of tissue damage, accumulate in the epineurium after nerve injury, contributing to neuropathic pain [29]. Depletion of circulating neutrophils, by using antibodies, attenuates the induction of thermal hyperalgesia after PSNL but does not alleviate the typical hyperalgesia at day 8 after injury, suggesting that endoneurial accumulation of neutrophils is related to the development of neuropathic pain [30]. Macrophages are significantly increased in neuropathic pain models [11, 31]. Moreover, systemic depletion of macrophages reduces mechanical hypersensitivity, suggesting their importance in the generation of neuropathic pain [32]. Furthermore, the role of Schwann cells in the generation of neuropathic pain has been confirmed [33]. In a chronic constriction injury (CCI) rat model, Schwann cells release erythropoietin (EPO), while injection of EPO may protect against neuropathic pain [34, 35]. CCI induces T cell infiltration, as well as mechanical allodynia and thermal hyperalgesia. These neuropathic pain symptoms are significantly reduced in rats lacking mature T cells, indicating that T cells are key immune cells in the development of neuropathic pain. There are two main T helper (Th) cell subtypes: Th1 cells and Th2 cells. Th1 cells produce proinflammatory cytokines and Th2 cells produce anti-inflammatory cytokines. Injection of these two types has opposite effects: Th1 cell injection enhances neuropathic pain symptoms, while Th2 injection reduces pain hypersensitivity [36].

A wide variety of experimental approaches have indicated that inflammatory mediators play crucial roles in neuropathic pain. Proinflammatory cytokines and chemokines have been associated with neuropathic pain and play critical roles in this condition by direct and indirect actions. Some of these factors can activate and sensitize nociceptors and thereby induce pain [37]. Furthermore, nerve injury produces proinflammatory cytokines: TNF-*α*, IL-1*β*, and IL-6 and inflammatory mediators: prostaglandin, serotonin, bradykinin, and histamine, as well as neurotrophic factors, such as nerve growth factor (NGF). On the other hand, some immune cells can produce and release anti-inflammatory cytokines and opioid peptides, such as *β*-endorphin, which may induce analgesia and alleviate pain [38]. Taken together, these studies indicate that several immune cell types and their mediators are implicated in the development of neuropathic pain [18].

In a neuropathic pain model, IL-1*β* expression is upregulated in the injured sciatic nerve, DRG, and spinal cord [39–41]. Interestingly, the injection of an IL-1*β*-neutralizing antibody was shown to alleviate the behavior related to neuropathic pain [40]. IL-1*β* functions to enhance excitatory synaptic transmissions and reduce inhibitory synaptic transmissions in the dorsal horn neurons [42]. It has also been reported that TNF-*α* was upregulated in a neuropathic pain model and this phenomenon was confirmed by specific inhibitor treatment [43, 44]. Xu and colleagues have suggested that TNF-*α* may be a biomarker for chronic neuropathic pain in patients with spinal cord injury (SCI) [45]. Furthermore, IL-6 is also a neuropathic pain-related proinflammatory cytokine. In a model involving partial ligation of the sciatic nerve, IL-6 expression was increased [46]. TNF-*α* only enhances excitatory synaptic transmissions, while IL-6 reduces inhibitory synaptic transmission at the same spinal cord level [42]. Additionally, NGF-overexpressing mice showed hypersensitivity [47]. Moreover, MCP1, also known as CCL2, is a major inflammatory chemokine that is specifically responsible for recruiting monocytes into the site of inflammation. After nerve damage,* MCP1* mRNA expression was significantly upregulated in the rat DRG [48]. In addition, intrathecal injection of MCP1 produces mechanical allodynia [48].

### 2.2. Regulation of Peripheral Nerve Excitability

Nerve injury increases expression of sodium channels in the DRG and around the terminal injury site of injured axons. Nav1.7 and Nav1.8 double-knockout mice normally develop neuropathic pain in PSNL animal model [49]. Nav1.7 is not required for oxaliplatin-induced neuropathic pain but is essential in CCI mice, suggesting that the neuropathic pain trigger affects the mechanism by which pain develops [50]. In addition, knockdown of Nav1.8 by means of siRNA alleviates mechanical allodynia in CCI rats [51].

Nav1.7 and Nav1.8 expression are also significantly increased in the lumber disc herniation model [52]. Nav1.3 is upregulated in the DRG after nerve injury, in line with the data showing that knockdown of Nav1.3 reduces pain in the neuropathic pain model [53, 54].

Drugs blocking sodium channels include lidocaine, mexiletine, phenytoin, carbazepine, oxcarbazepine, lamotrigine, and TCAs. These drugs, which are nonselective for channel subtypes, can alleviate neuropathic pain by interfering with spontaneous ectopic discharges but also have many side effects [55].

Voltage-gated calcium channels (Cav) also play a role in neuropathic pain development. T-type channel current density significantly increases after nerve injury, and inhibitors of this type of channel can reduce excitability in a neuropathic pain model [56, 57]. Mice lacking the N-type channel show reduced responses to mechanical and thermal stimuli in neuropathic pain models [58].

Ligand-gated ion channels also have crucial roles in terms of the onset of neuropathic pain. Interestingly, agonists and antagonists of transient receptor potential vanilloid member 1 (TRPV1) are both effective in neuropathic pain alleviation. TRPV1 agonists release transmitters and induce cation influx into the nerve, as well as receptor desensitization [59]. Topical formulations of high-dose capsaicin have been shown to be efficacious in a number of neuropathic pain conditions, including Phase 3 studies in postherpetic neuralgia patients [60]. It has been reported that antagonists that block TRPV1 signal transduction can reverse heat-related hyperalgesia in sciatic nerve ligation in mice [61]. After chronic compression of the DRG, mechanical allodynia was enhanced by injection of a TRPV4 agonist but reversed by its antagonist [62]. Antagonists of NMDA receptors, which are subtypes of the glutamate receptor, are third-line treatments for neuropathic pain and can reduce pain in animal models but have many side effects, including sedation, confusion, and motor incoordination [63].

### 2.3. G Protein-Coupled Receptors (GPCRs)

G protein-coupled receptors (GPCRs) comprise the largest superfamily of transmembrane receptors and transduce extracellular stimuli into intracellular responses. Targeting GPCRs is highly successful, so that 30% of drugs target GPCRs for conditions including allergies, hypertension, migraine, asthma, stroke, and pain [64]. GPCRs and their ligands play a number of important roles in the modulation of acute and chronic pain, including neuropathic pain [65].

#### 2.3.1. GABA Receptors

Gamma-aminobutyric acid (GABA) is a widely distributed inhibitory neurotransmitter, also found in the spinal cord. It is well-known that hypofunction of GABAergic tone leads to development of neuropathic pain in animal models [66]. Treatment with GABA agonists reduces central neuropathic pain behavior and neuronal hyperexcitability after SCI. Injection of GABA after SCI attenuated mechanical allodynia and hyperexcitability of the spinal dorsal horn neurons. This reduction is regulated by both GABAA and GABAB receptors. Moreover, bicuculline, a GABAA receptor antagonist, induces hyperexcitability and pain behavior in normal rats [67]. The number of GABAergic interneurons is also decreased in a neuropathic pain model [68, 69].

#### 2.3.2. Bradykinin Receptors

Bradykinin (BK), an inflammatory mediator, is a vasodilator and is known to induce neuropathic pain by binding to the BK1 and BK2 receptor. Several studies have shown the participation of kinins and their receptors in neuropathic pain development [90]. Nerve injury induces an increase in BK1 receptor mRNA in the spinal cord and sciatic nerve and also produces mechanical allodynia and hyperalgesia. The BK1-knockout mice show reduction in both allodynia and hyperalgesia in a neuropathic pain animal model. Inhibition of BK1 and BK2 reverses the effect of dynorphin A, an endogenous opioid peptide, inducing persistent neuropathic pain [91].

#### 2.3.3. Opioid Receptors

Most opioids are used as second- or third-line analgesics that may provide reasonable analgesia to some neuropathic pain [92]. Because opioid treatment may require relatively higher doses, opioid-related adverse reactions are common. Morphine is an opioid analgesic and reduces neuropathic pain, but the mechanisms underlying this reduction are unclear [93]. There are four types of known opioids receptors: delta (DORs), kappa (KORs), and mu-opioid receptors (MORs) and opioid receptor like-1 (ORL1). DOR-knockout mice showed increased neuropathic pain, implying that endogenous delta opioid activity reduces chronic pain [94]. TCAs alleviate allodynic effects in a neuropathic pain model; however, in DOR-knockout mice, the efficacy of these drugs was reduced [95]. Antidepressant drugs are still effective in MOR-knockout animals, suggesting that these drugs are regulated by DOR rather than MOR [96]. KORs are also not necessary for the effect of TCAs against neuropathic allodynia. These data indicate that DOR is the only opioid receptor that is necessary for the antiallodynic action of antidepressants [97]. In addition, ORL1 receptor agonist relieves thermal hyperalgesia after nerve injury [98].

#### 2.3.4. Histamine Receptors

Histamine is an organic nitrogenous compound involved in local immune responses as well as in regulating physiological function. As mentioned above, Nav1.8 upregulation in primary afferents plays a critical role in the development and persistence of neuropathic pain, although the mechanisms underlying this upregulation are not fully understood. Histamine increases Nav1.8 expression in primary afferent neurons [99], while histamine receptor antagonists suppress mechanical allodynia in neuropathic rats [27].

#### 2.3.5. Prostaglandin E2 Receptors

Prostaglandin E2 (PGE2) is a well-known mediator of inflammation and pain and plays a pivotal role in nociceptive processing and sensitization in the spinal cord. After nerve injury, the pain mediator, cyclooxygenase 2 (COX2), and its end product, PGE2, are persistently upregulated in invading macrophages. Nervous tissue damage induced PGE2 contributes to upregulation of BDNF in DRG neurons, leading to neuropathic pain [100].

#### 2.3.6. CCR2

Among GPCRs, chemokines associated with the immune system are remarkable neuropathic pain modulators. Chemokines constitute a large family of relatively low-molecular weight proteins, that is, chemoattractant cytokines controlling immune cell trafficking. MCP1 (CCL2) is a member of the CC chemokine family that specifically attracts and activates monocytes to sites of inflammation. This chemokine is absent from the normal CNS and is found after inflammation and pain, including neuropathic pain, development. CCR2, the receptor for MCP1, is expressed selectively on cells of the monocyte/macrophage lineage in the periphery and in neurons and astrocytes in the brain. Peripheral nerve injury can result in a disruption of the blood spinal cord barrier (BSCB), allowing influx of peripheral immune cells, which is mediated by MCP1. Additionally, activated microglial cells express CCR2 and modulate the CCR2-CCL2 interaction between injured primary afferent fiber terminals and dorsal horn microglia [101]. Interestingly, spinal microglia activation occurs during the early phase of neuropathic pain, suggesting that microglia may be important for initiation of neuropathic pain, while astrocytes are important for its maintenance. In addition, one study has demonstrated that CCL2/CCR2 signaling in the DRG and spinal cord is involved in neuropathic pain via distinct mechanisms [102]. Nucleus pulposus-induced mechanical allodynia is attenuated by treatment with the CCR2 antagonist RS504393 in radicular neuropathic pain. RS504393 decreased lipopolysaccharide-evoked upregulation of the CCL2 and CCR2 expression and protein level in primary microglial cell cultures [103]. Mice lacking CCR2 showed substantially less hypersensitivity to mechanical stimulation after nerve injury but showed a normal response in acute pain [101]. Currently, CCR2 antagonists are in clinical trials and may be promising medicines for neuropathic pain patients in the future.

## 3. Natural Products and Compounds Ameliorating Neuropathic Pain

Natural products and compounds derived from them have shown analgesic effects in neuropathic pain models, by various modes of action (Table 1).

### 3.1. Food-Derived Compounds against Neuropathic Pain

A number of studies have indicated that neuroinflammation plays an important role in the induction and maintenance of chronic pain. Considering this importance, decreasing neuroinflammation through regulation of anti-inflammatory and inflammatory mediators is promising for treating neuropathic pain. According to a systematic review of natural products evaluated in neuropathic pain models, the five most researched compounds are as follows: flavonoids (28%), terpenes (17%), alkaloids (14%), phenols (10%), and carotenoids (10%) [104]. Food-derived compounds alleviating neuropathic pain are described in Table 1. Briefly, capsaicin is a well-known TRPV1 agonist. Unusually, capsaicin induces pain by a single treatment but relieves pain with repeated injections [105]. Intrathecal capsaicin injection attenuates thermal hyperalgesia but does not reduce mechanical allodynia in nerve-injured rats [71].

Palmitoylethanolamide (PEA), an endogenous fatty acid amide, has been shown to perform various biological functions related to chronic and neuropathic pain and inflammation in clinical trials. PEA is an endogenous modulator found in food, such as eggs and milk, and no serious side effects to this treatment have been reported. PEA treatment was shown to relieve both thermal hyperalgesia and mechanical allodynia by regulating cannabinoid receptor 1 (CB1), peroxisome proliferator-activated receptor *γ* (PPAR*γ*), and TRPV1 receptors in neuropathic pain model mice [82]. In clinical studies, PEA was shown to alleviate neuropathic pain regardless of age, sex, and the pain trigger [106].

Zerumbone, a bioactive compound derived from* Zingiber zerumbet* was demonstrated to exert antiallodynic and antihyperalgesic effects in a CCI mouse model [89]. Additionally, most available evidence suggests that curcumin alleviates mechanical allodynia and thermal hyperalgesia in a CCI injured model [107, 108]. This compound modulates the dopamine receptor and downregulates p300/CBP HAT activity, reducing expression of* BDNF* and* COX2* in a neuropathic pain rat model [109].

Dehydrocorybulbine (DHCB) is isolated from* Corydalis yanhusuo*, a traditional pain relief plant. Synthetic DHCB has efficacy in acute pain, inflammatory pain, and neuropathic pain, without side effects, such as sedation. These antinociceptive effects are reversed by a dopamine D2 agonist in the tail-flick test. DHCB exhibits affinities to dopamine receptors and displays its highest affinity to the D2 receptor [76]. Interestingly, both dopamine D2 receptor agonists and antagonists have been reported to have analgesic properties [110, 111].

*β*-Caryophyllene (BCP) is natural selective agonist of the peripherally expressed cannabinoid receptor 2 (CB2), an important receptor in neuropathic pain, and is found in many spices and food plants. Administration of BCP attenuated thermal hyperalgesia and mechanical allodynia and reduced spinal neuroinflammation in mouse models of inflammatory and neuropathic pain [72].

Huperzine A (HUP-A), a naturally occurring* Lycopodium* alkaloid, isolated from* Huperzia serrata*, exerts potent reversible inhibition on acetylcholinesterase and NMDA receptors, alleviating neuropathic pain, without drug tolerance and dependence [112].

Quercetin has been described as an antioxidant, antinociceptive, and anti-inflammatory compound and has preventive effects against oxaliplatin-induced painful peripheral neuropathy [113].

Berberine is a plant alkaloid that is derived from various plants and has been reported to show multiple pharmacological properties of use in several conditions, including neuropathic pain. Berberine exerts an antidepressant effect by modulating dopamine. Berberine could reduce cold and mechanical allodynia, induced by nerve injury and diabetic neuropathy [70, 114].

Chlorogenic acid (CGA) is a natural organic phenolic compound that is found in many plants, fruits, and vegetables, including Yerba mate. CGA has beneficial bioactivities and strong anti-inflammatory effects. In addition, administration of CGA prevented the development of mechanical hyperalgesia and attenuated histopathological changes induced by nerve injury [115].

Additionally, isoflavones are phytoestrogen used for treating hormone dysregulation like menopause and effective in diverse diseases. Genistein, a soy isoflavone, ameliorates painful neuropathy via multiple mechanisms, regulating estrogen receptor-*β*, and antioxidant and anti-inflammatory activities [77].

In streptozotocin-induced diabetic mice, treatment with lycopene, a carotenoid mostly found in tomatoes, attenuated neuropathic pain by inhibiting nitric oxide and TNF-*α* release [116]. In addition, treatment with lycopene notably prevented mechanical hypersensitivity and downregulated CX43 expression in the spinal dorsal horn [79].

Naringin, a flavonoid abundant in citrus fruits, such as grapefruits, has been reported to possess anti-inflammatory properties. Naringin reversed the pain response in STZ-induced diabetic rats by altering expression of endogenous biomarkers [80].

Moreover, there are many studies showing that omega-3 polyunsaturated fatty acids (PUFAs) have positive effects on mood, as well as analgesic effects [117–119]. Omega-3 is effective against inflammatory pain and neuropathic pain. This compound does not interact with the pharmacodynamics or pharmacokinetics of commonly used analgesic drugs [120] and can therefore be used concomitantly with conventional analgesics. In addition, resolvin E1 derived from omega-3 also has analgesic effects in neuropathic pain [86].

Resveratrol is a natural polyphenolic compound with anti-inflammatory, cell growth-modulatory, and anticarcinogenic properties and is used to treat cardiovascular diseases, cancers, and aging. Injection of resveratrol increased the silent information regulator 1 (SIRT1) and decreased acetyl-histone H3, alleviating neuropathic pain [87].

In addition,* Harpagophytum procumbens*, also known as Devil's Claw, is a plant species that has widespread medicinal uses, for treating fever, malaria, indigestion, and pain.* H. procumbens *extracts significantly reduced nerve injury-induced mechanical allodynia [121].

Furthermore,* Ginkgo biloba* extract is known for its beneficial effects on brain functioning. The administration of* G. biloba* extract attenuates mechanical and cold allodynia in an animal model, suggesting it may be promising target for neuropathic pain. However, sedation and motor disturbance were observed with high-dose treatments [122].

### 3.2. CCL2/CCR2 Downregulation by Natural Compounds

CCL2 and CCR2 are involved in the pathology of a number of diseases, including rheumatoid arthritis, multiple sclerosis, atherosclerosis, asthma, and neuropathic pain [123]. Recently, pharmaceutical companies have taken an interest in the discovery and development of CCR2 antagonists for the treatment of neuropathic pain and other diseases. We and others have also attempted to find natural products with CCR2 antagonistic effects, with a view to developing new drugs or functional foods against various diseases. Many studies have indicated that natural compounds can reduce CCL2 expression in diverse models. The diallyl disulfide found in garlic inhibits TNF-*α*-induced CCL2 release* in vitro* by suppressing CCL2-CCR2 signaling [124]. Similarly, other natural compounds, including caffeic acid, CGA, berberine, amorfrutin A, and curcumin, decrease CCL2 expression [125–129]. There are very few studies about natural compounds that alleviate neuropathic pain by inhibiting CCL2-CCR2 binding, although some natural compounds have been shown to decrease the expression of CCR2 in disease models. Therefore, the compounds or plant extracts and other agents that can reduce expression of CCL2* in vitro* and* in vivo* should be studied further to determine whether these natural compounds have potential CCR2 antagonistic effect or can reduce the CCL-CCR binding affinity in neuropathic pain models.

## 4. Conclusion

Recently, natural products and their constitutive compounds have come to be considered as a new direction for development of analgesics and functional foods to resolve pain and pain-related problems. Most of the evidence suggests that GPCRs are the most promising potential targets for pain therapeutics, and CCR2, among the GPCRs, plays a particularly crucial role in the early phase during the onset of neuropathic pain. Overactivated microglia can also trigger neuropathic pain via CCR2-related signaling, resulting in neuroinflammation. Therefore, a CCR2 antagonist may have an effect as a neuropathic pain reliever. Further studies are needed to identify natural products that can inhibit CCL2-CCR2 binding. Although toxicological and pharmacological investigations are needed to clarify the distinctive mechanism of action and evaluate their safety in humans, natural products and their compounds may be useful in the treatment of neuropathic pain.

## Figures and Tables

**Table 1:** Food-derived compounds alleviating neuropathic pain *in vivo*.

Food compound	Source	Target	Site	Animal model	Reference
Berberine	*Berberis vulgaris*	NF-*κ*B	PNS	CCI	[70]
Capsaicin	Hot chili peppers	TRPV1	PNS	CCI	[71]
*β*-Caryophyllene	*Origanum vulgare* L.	CB2	Spinal cord	PNL	[72]
Chlorogenic acid	*Ilex paraguariensis*, coffee	GABA receptor, NPY	spinal cord, DRG	SNI, CCI	[73, 74]
Curcumin	*Curcuma longa*	DR, CREB, phospholipase C	cerebral cortex and cerebellum	Diabetic model	[75]
DHCB	*Corydalis ambigua *var*. amurensis*	DR	Brain	SNL	[76]
Genistein	Soy	ER*β*, NOS, NF-*κ*B	sciatic nerve, DRG, spinal cord, thalamus	CCI	[77]
Huperzine A	*Huperzia serrata*	mAChRs	PNS	CPN	[78]
Lycopene	Tomato	Cx43	spinal astrocytes	PNL	[79]
Naringin	Grape	inflammatory mediators, apoptosis	neural cell	Diabetic model	[80]
Omega-3	Fish oil	p38 MAPK	Spinal cord	SCI	[81]
PEA	Eggs and milk	CB1, TRPV1, PPAR*γ* receptors	Spinal cord	CCI	[82]
Phlorotannins	Sea weed	Inflammatory mediators	DRG	SNI	[83]
Quercetin	Red kidney beans, capers	OPR	PNS, CNS	Diabetic model, CCI	[84, 85]
Resolvin E1	Omega-3 polyunsaturated fatty acid	Microgliosis, TNF-*α*	microglia	SNL	[86]
Resveratrol	Grapes, nuts, berries	SIRT1	Spinal cord	CCI	[87, 88]
Zerumbone	*Zingiber zerumbet*	TRPV1, TRPA1	DRG	CCI	[89]

DHCB: dehydrocorybulbine, PEA: palmitoylethanolamide, NF-*κ*B: nuclear factor kappa-light-chain-enhancer of activated B cells, TRPV1: transient receptor potential vanilloid 1, CB2: cannabinoid receptor type 2, GABA: gamma-aminobutyric acid, NPY: Neuropeptide Y, DR: dopamine receptor, CREB: cAMP response element-binding protein, ER*β*: estrogen receptors, NOS: nitric oxide synthase, mAChRs: muscarinic acetylcholine receptors, cx43: connexin 43, p38 MAPK: p38 mitogen-activated protein kinases, PPAR*γ*: peroxisome proliferator-activated receptor, OPR: opioid receptor, TNF-*α*: tumor necrosis factor-*α*, SIRT1: Sirtuin 1, TRPA1: transient receptor potential ankyrin 1, PNS: peripheral nervous system, DRG: dorsal root ganglion, CNS: central nervous system, CCI: chronic constriction injury, PNL: partial nerve ligation, SNI: spared nerve injury, SNL: spinal nerve ligation, and CPN: Common Peroneal Nerve.
